# Supplementary material for: Evaluation of Machine Learning and Traditional Statistical Models to Assess the Value of Stroke Genetic Liability for Prediction of Risk of Stroke Within the UK Biobank
Source: Healthcare (Basel). 2025 Apr 26;13(9):1003. doi: 10.3390/healthcare13091003 (PMC12071721; doi:10.3390/healthcare13091003)
Supplement: Supplementary file 1 [file healthcare-13-01003-s001.zip › healthcare-3501066-supplementary.pdf]

Supplementary Information for

Evaluation of Machine Learning and Traditional Statistical Models to Assess the Value of Stroke Genetic Liability for Prediction of Risk of Stroke within the UK Biobank.

Gideon MacCarthy and Raha Pazoki

Supplementary Table S1: Overview of previous studies investigating the effect of genetic liability on risk of stroke.

Author, in-text citation	Genetic liability characteristics	Stroke outcome	Mode name	Measure of risk	Prediction performance	Ethnicity
Myserlis et al., 2023 [22]	metaGRS (Combined 21 GRS)	hemorrhage	Coxph	HR = 1.15	C-index Increased from 68.9% to 69.5% when metaGRS was added to the model.	European ancestry
Rutten-Jacobs et al., 2018 [23]	90-SNPs GRS for any stroke	All Stroke	Coxph	HR = 1.35	N/A	European ancestry
Abraham et al., 2019 [25]	metaGRS (Combined 19 GRSs)	Ischemic	Coxph	HR = 1.26	N/A	European ancestry
Papadopoulo et al., 2024[40]	28-SNPs GRS and conventional risk factors	Ischemic	ML	NA	XGBoost with ROC of 63.1%.	European Ancestry with AF
Wang et al., 2023[41]	Selected conventional risk	Hemorrhage	Coxph and ML	NA	RF (AUC = 87.5%)	United states

	factors				vs Coxph(AUC = 76.1%)	
Cárcel-Márquez et al., 2022 [73]	93-SNPs GRS and Selected conventional risk factors	cardioembolic	MTAG	NA	AUC=94.7% without GRS AUC = 95.0% when GRS was added.	European ancestry
Jung et al., 2018 [74]	16-SNPs GRS and selected conventional risk factors.	stroke	Coxph	NA	AUC= 67%	Korean

- *MTAG:Multitrait Analysis of Genome Wide Association Study*

Supplementary Figure S1: Overview of the process to create genetic liability for stroke within the UK Biobank.

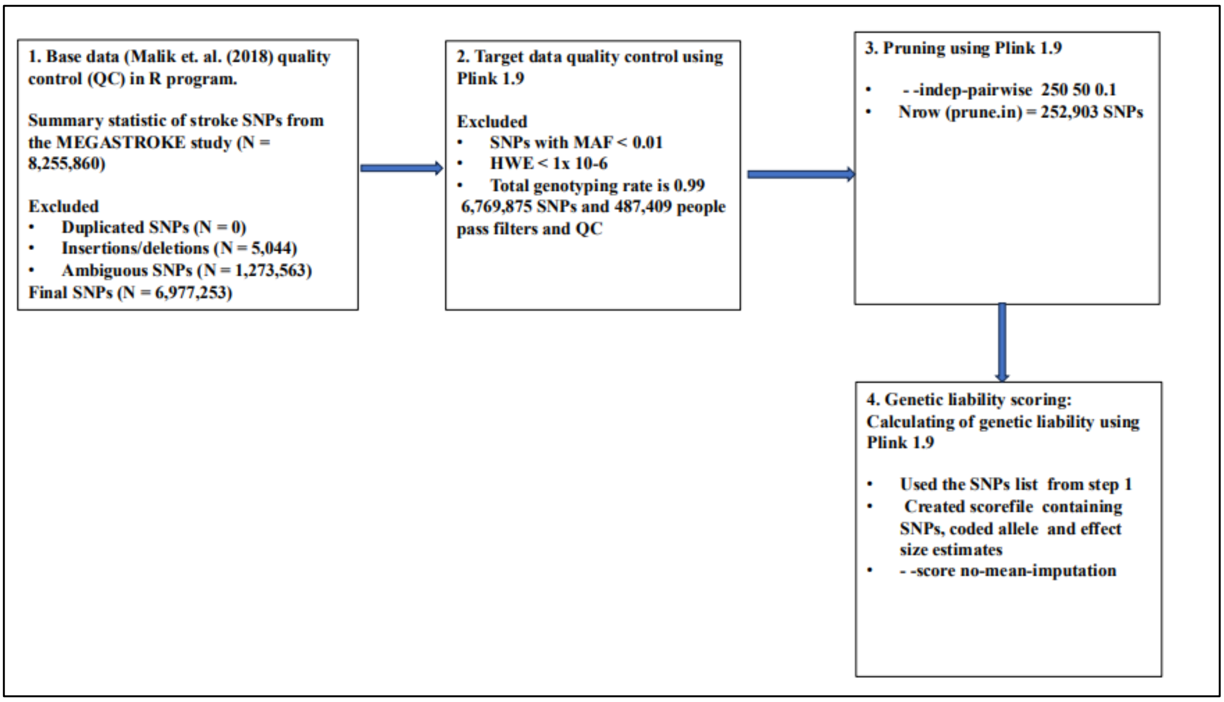

Genetic liability calculation process: SNP: Single nucleotide polymorphism; **MAF**: Minor allele frequency; **LD**: Linkage disequilibrium; **HWE**: Hardy-Weinberg Equilibrium. The SNPs were pruned with plink command - -indep-pairwise window size = 250, step size = 50, r^2 = 0.1. **Base data**: SNP list from Malik et. al. (2018), **Target data**: genotype data in plink binary format.

Supplementary Figure S2: Workflow diagram illustrating the inputs and output for machine learning **models.**

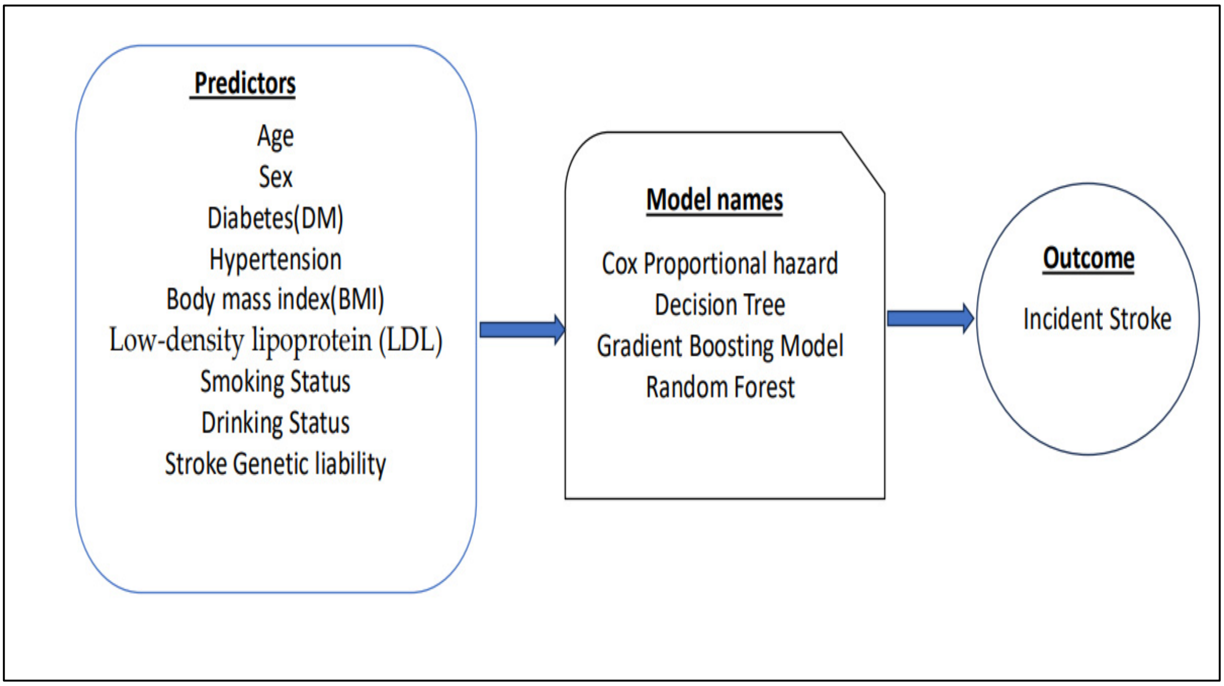

Supplementary Figure S3: Overview of the modeling and predictions process for using machine learning and genome-wide genetic liability for prediction of risk of stroke within the UK Biobank.

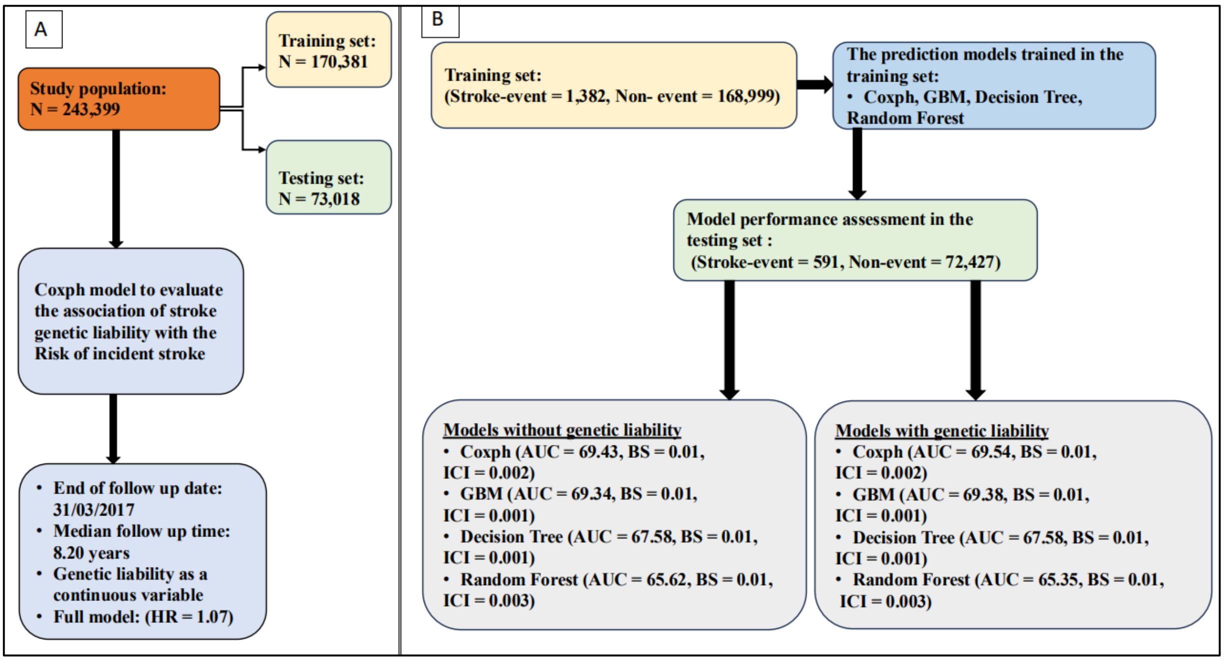

Stroke risk prediction model creation and performance evaluation: Coxph: Cox proportional hazard, GBM: Gradient Boosting model, HR= Hazard ratio, AUC: Area under the curve, BS: Brier score, ICI: Integrated calibrated index. Panel A: Assessing the association between genetic liability and incident stroke. Panel B: Stroke risk prediction modeling and performance evaluation.

Supplementary Figure S4: Schoenfield test results of full Cox proportional hazard model.

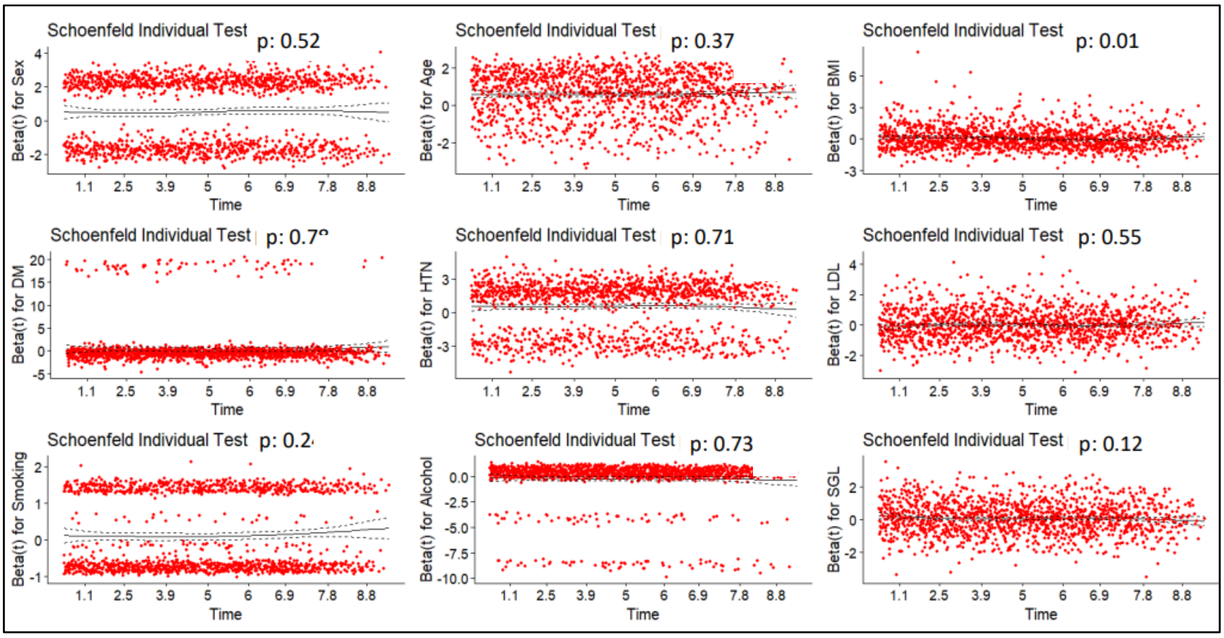

The figure illustrates an assessment of the proportional hazard (PH) assumption using the global Schoenfield test that assesses proportional hazard assumption for all covariates from a multivariate model. The test indicated a p-value of 0.14 indicating no significant time-dependent effect on the covariates jointly. If P-value > 0.05, the test fails to reject the null hypothesis i.e. the PH assumption would hold for the overall model and covariates have a consistent effect over time. The individual Schoenfield test for BMI indicates that variable does not have a consistent effect over time. Thus, BMI is adjusted within all the Cox models in the study. BMI: Body Mass Index, LDL: Low-density lipoprotein cholesterol, SGL: Stroke genetic liability

Supplementary Figure S5: Roc plot of coxph models

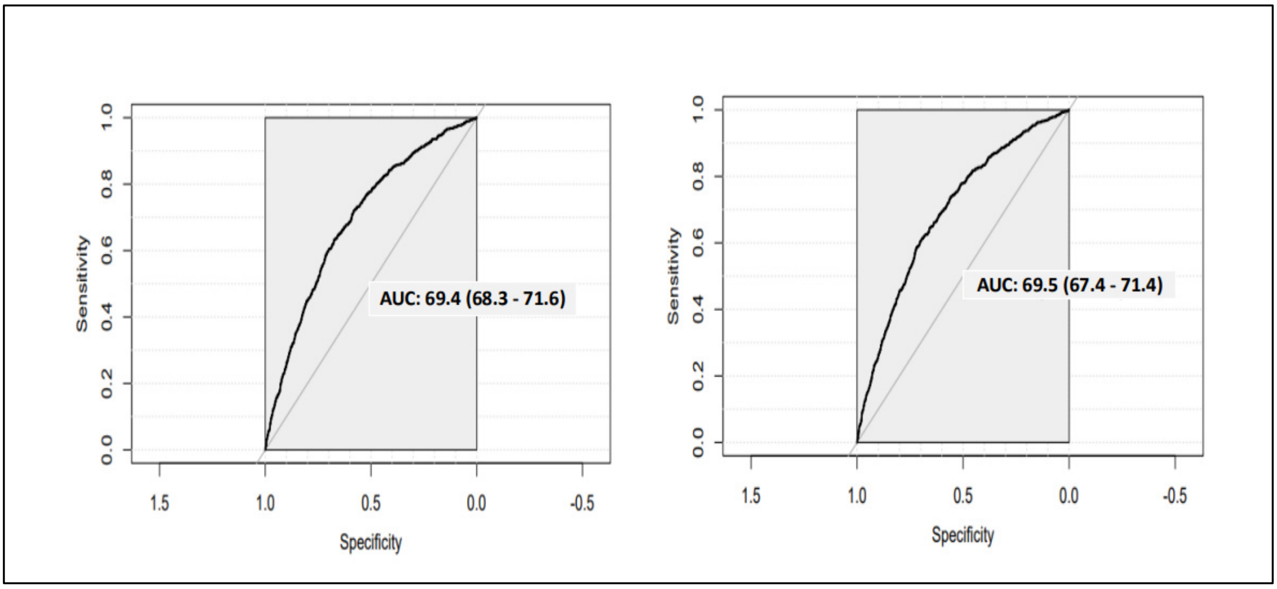

Left panel: Model using conventional risk factors. **Right panel:** Model using conventional risk factors and stroke genetic liability.

Supplementary Figure S6: Roc plot of Gradient boosting models

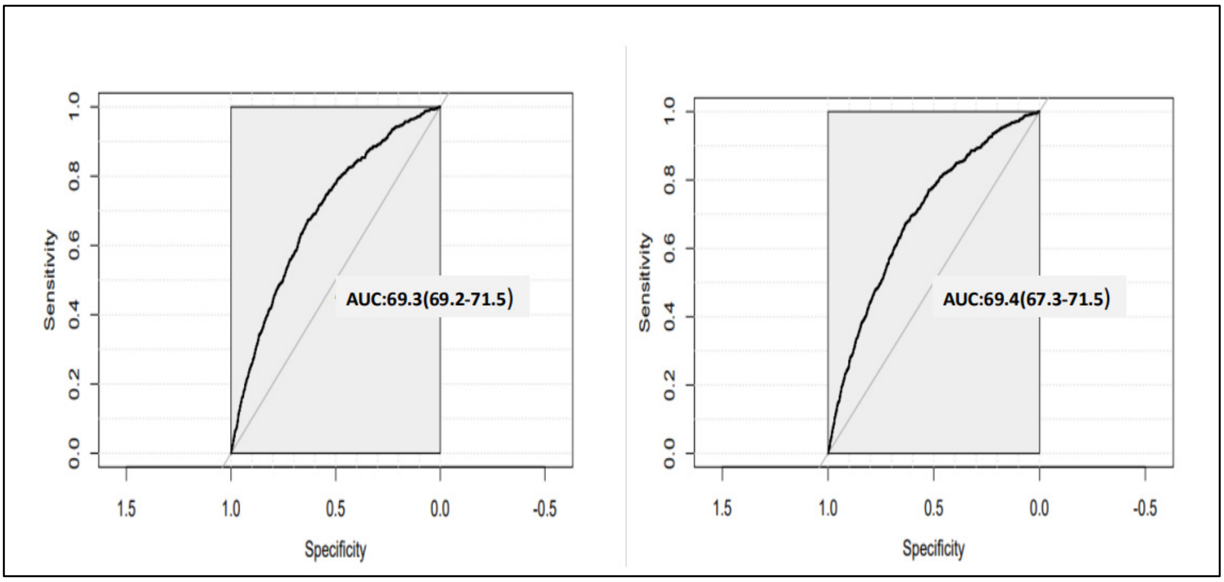

Left panel: Model using conventional risk factors. **Right panel:** Model using conventional risk factors and stroke genetic liability

Supplementary Figure S7: Roc plot of decision tree models (using pruning)

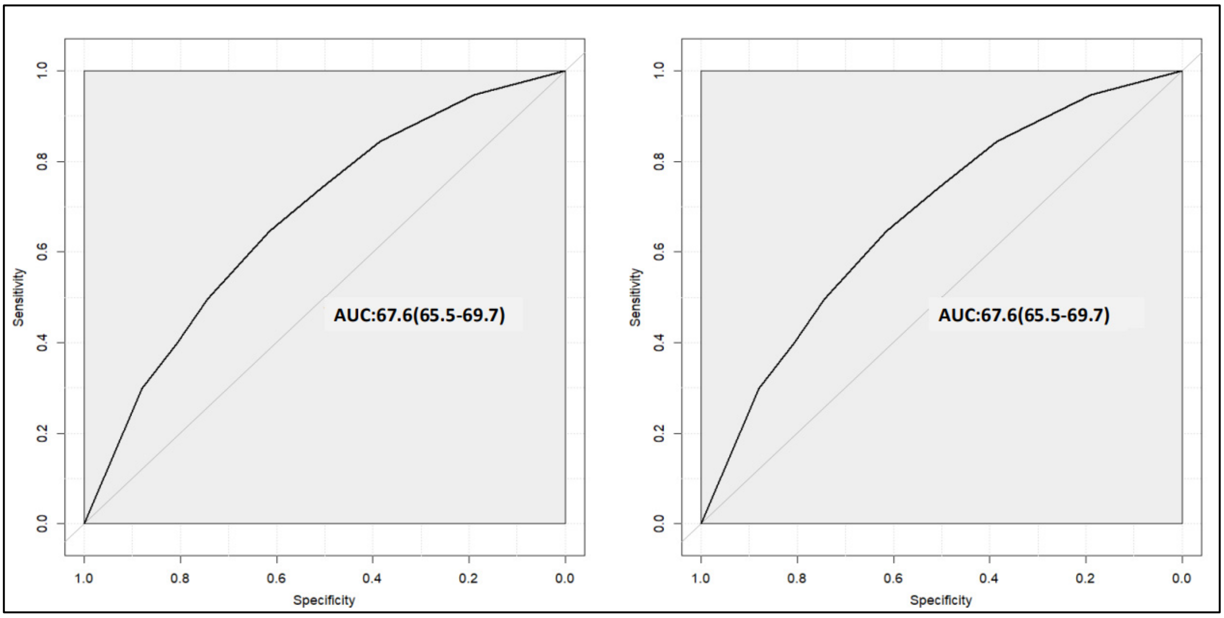

Left panel: Model using conventional risk factors. **Right panel:** Model using conventional risk factors and stroke genetic liability

Supplementary Figure S8: Roc plot of decision tree models (without pruning)

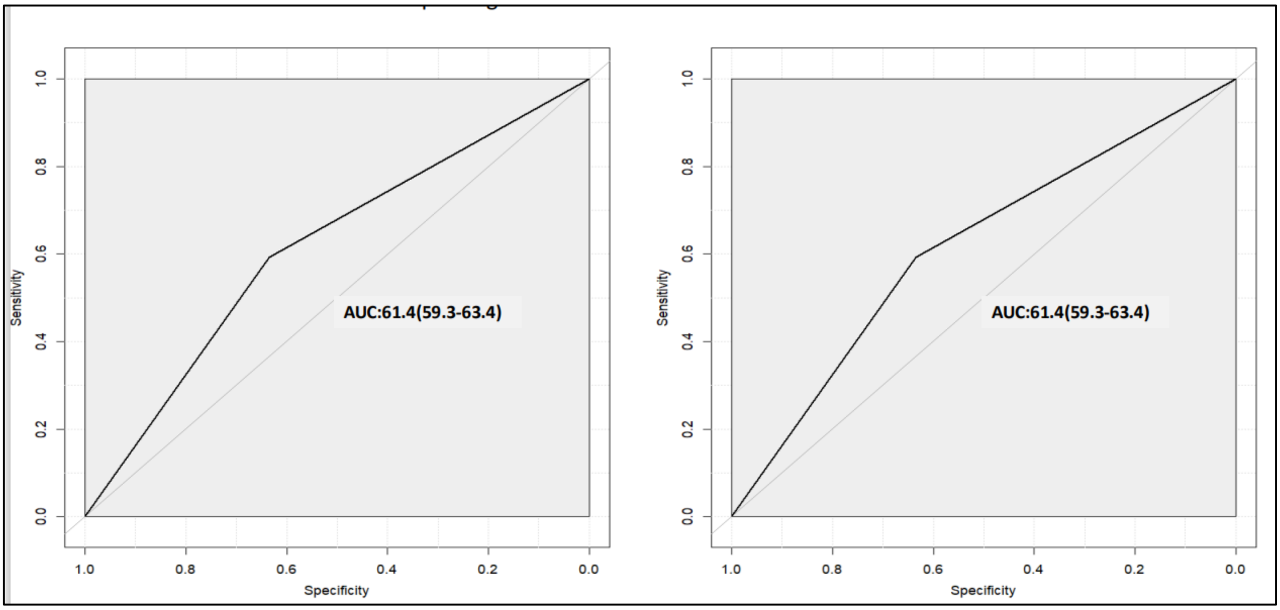

Left panel: Model using conventional risk factors. **Right panel:** Model using conventional risk factors and stroke genetic liability

Supplementary Figure S9: Roc plot of Random Forest models

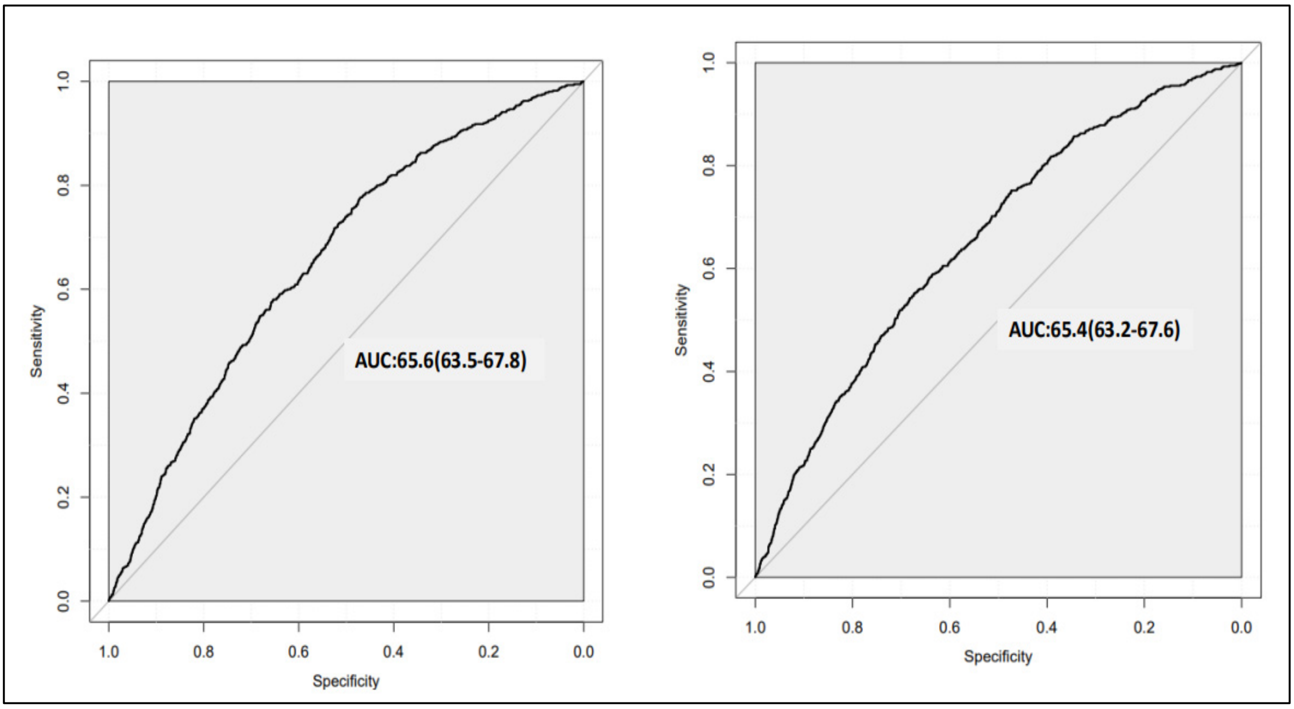

Left panel: Model using conventional risk factors. **Right panel:** Model using conventional risk factors and stroke genetic liability
